# Gaining super control: Psychoeducational group intervention for adolescents with mild intellectual disability and their parents

**DOI:** 10.1177/1744629521995373

**Published:** 2021-03-15

**Authors:** Stine Ericson, Marianne Winge Hesla, Kristine Stadskleiv

**Affiliations:** 155272 Oslo University Hospital, Norway; 155272 Oslo University Hospital, Norway; 155272 Oslo University Hospital, Norway; University of Oslo, Norway

**Keywords:** adolescence, group intervention, intellectual disability, psychoeducation, transition

## Abstract

Adolescents with intellectual disability experience psychological and social challenges in their transition to adulthood. Knowledge about the diagnosis and insight into own strengths and difficulties can help them manage the limitations and barriers they face, but suitable interventions with this purpose are scarce. The present paper presents a psychoeducational group intervention, The Super Control Project, for adolescents (15–17 years old) with mild intellectual disability (n = 23) and their parents. In a pre-post design, adolescent outcome data was obtained through teacher and parent questionnaires, and interviews with the adolescents. Parents and adolescents also evaluated the interventions’ usefulness. Results indicated positive impact on participants’ understanding of the diagnosis, managing of everyday challenges, and social networking. The intervention seemed to fit the participants’ needs and abilities. The study encourages further implementation and rigorous evaluation.

## Introduction

Adolescence is generally considered to be a time of preparation for adulthood. Key issues in the psychosocial development at this stage are identity formation, increasing independence, and individuation from the family (Erikson, 1994; von Tetzchner, 2018). All adolescents have a challenging time developing adult skills, but adolescents with intellectual disability need closer follow-up, facilitation, and more systematic learning experiences to acquire the skills required for adulthood (Parmenter et al., 2016). For young people with intellectual disability, adolescence is known to represent increased risk for social isolation, mental health problems, and greater reliance on family (Merrells et al., 2019). Prevalence of any psychological disorder in children and adolescents with intellectual disability is between 30 and 50% (Einfeld et al., 2011). Many experience loss of friendships related to the progressively decreasing presence and participation in the regular classroom throughout their secondary schooling (Carter and Hughes, 2005). Increased autonomy is often more difficult to achieve for these youngsters because of their reliance on parents (Marshall et al., 2018). Nevertheless, most adolescents with disabilities desire to have developmental experiences and social opportunities similar to their typical developing peers (LoConto and Dodder, 1997; Maxey and Beckert, 2017).

Approximately 85% of all individuals with intellectual disability have a mild level of disability (Carr and O’Reilly, 2016). Young adults with mild intellectual disability do possess the potential to live at least partially independent lives, and to work if they receive appropriate life skills instruction. However, without this preparation they often fail to obtain and hold jobs (Parmenter et al., 2016). Limitations in their basic life skills, together with environmental barriers, will often limit their quality of life.

The diagnosis of intellectual disability is associated with significant stigma (Todd and Shearn, 1997). Managing the stigma of having intellectual disability is a key issue for those diagnosed and may lead to difficulty obtaining a positive self-concept (Kenyon et al., 2014) and the use of maladaptive coping strategies like denial (McVittie et al., 2008). Since individuals with intellectual disability form a heterogeneous group, it is perhaps difficult for adolescents with mild intellectual disability to accept the diagnosis when comparing themselves to others who might not only be substantially more cognitively impaired but may in addition have significant motor or sensory impairment - thereby making the disability more visible. For many people with mild intellectual disability, the diagnostic evaluation is carried out during early school age (Carr and O’Reilly, 2016), at a time where parents often consider it too early to inform their child about the diagnosis. This implies that dissemination of diagnostic information in many cases is left to the parents to handle on their own.

Jones et al. (2014) examined parent reports of conversations about disability and experiences of being different in families of adolescents with intellectual disability. Most parents seemed to grapple with what to say and when to say it. Much of the communication reported by parents appeared to be reactive (for example as response to social exclusion), as opposed to proactive. However, if parents do not speak with their adolescents about the disability, either out of a desire to protect them or because they perceive the disability as “bad,” adolescents may adopt the belief that there is something “wrong” with them. This lack of communication may cause the adolescent to not develop an adequate explanatory framework for their experiences, and can contribute to parents inadvertently increasing the stigma. The study highlights the parents’ struggle to explain the disability to their adolescents, as well as the importance of promoting proactive conversations between parents and adolescents.

Raising a child with a disability brings with it extraordinary challenges. The distress frequently experienced by these parents has been thoroughly documented. Parents of children with intellectual disability are at risk of financial, physical and psychological problems. Specifically, there is an increased risk of depression, and this appears to be related to an accumulation of stress and insufficient social support (Emerson, 2003; Gallagher et al., 2009; Hastings and Beck, 2004; Picard et al., 2014).

Adolescents with intellectual disability and their parents face challenges beyond those experienced by most families, and therefore need psychological support. Psychoeducation is among the most effective of the evidence-based practices that have emerged in both clinical trials and community settings (Lukens and McFarlane, 2004). It is a professionally delivered treatment that combines psychotherapeutic and educational interventions, and incorporates both illness-specific information and tools for coping and mastery. The central premise is that, the more knowledgeable the patients and caregivers are, the more positive the outcomes will be for all. It has long been applied for treatment of mental health problems, but has a broad potential for use in many kinds of illnesses and varied life challenges (Chiquelho et al., 2011; Dixon et al., 2000; Snead et al., 2004). The primary focus in previous interventions aimed at intellectual disability, both in terms of psychoeducational as well as other therapeutic approaches, has been on reducing parental stress or the adolescents’ challenging behavior through educating parents (Hastings and Beck, 2004; Schultz et al., 1993; Singer et al., 2007).

Interventions that go beyond alleviating parental stress or challenging behavior in adolescents are rare. However, we found one study which evaluated the effectiveness of a psychoeducational program compared to regular self-help support group for parents of adolescents with intellectual disability (Picard et al., 2014). The parents enrolled in the study had adolescents (n = 25) with all levels of disability (mild 31%; moderate 46%; severe 23%). The program consisted of 10 weekly information sessions of two-hour duration, covering topics related to intellectual disability. No significant differences were seen in the outcome measures of parental stress, depression, and anxiety between participants in the two groups, except that participants in the psychoeducational group rated the perceived impact of participation in the study somewhat higher. Participation in the psychoeducational program appeared to have given the parents access to more support, perhaps because in those sessions, they either learned of the existence of new resources or became motivated to take steps required to obtain more support. Other studies have also pointed out that providing parents of children with neurodevelopmental disabilities with relevant information in a group setting where they meet other parents, can contribute to network building, social support, and positive adaptation through creating a sense of empowerment and enhancing coping strategies (DaWalt et al., 2018; González-Fraile et al., 2019; Russell et al., 1999; Snead et al., 2004).

For adolescents with intellectual disability, psychoeducation implies learning about the diagnosis and, as part of that, how to cope with the associated stigma. Reactions to diagnosis have mostly been explored from the perspective of parents and professionals, but there are a few studies of adults with intellectual disability which suggest that learning about the diagnosis can trigger negative emotions and can take time to come to terms with (Kenyon et al., 2014; Monteleone and Forrester-Jones, 2017). As far as we have been able to ascertain, there are no previous studies on psychoeducational group intervention for adolescents with intellectual disability, nor any studies on adolescents' reactions to the diagnosis. Having knowledge about one’s diagnoses is, however, fundamental from a human rights perspective. The diagnosis is associated with rights for practical assistance, workplace interventions and financial support in many countries (Vornholt et al., 2018), and securing this can be vital for quality of life. Furthermore, having knowledge about why learning new skills and facts are more challenging than for peers, and that it is not related to lack of effort and motivation, can be of great importance for a person’s self-esteem and feelings of self-worth. The health care system is particularly suited for conveying information about diagnoses and practical implications of these and can thus play an important role in promoting proactive conversations between parents and adolescents.

### Aims

The purpose of this article is twofold; Firstly, to describe, and secondly to evaluate a psychoeducational group intervention, the Super Control Project, for adolescents with mild intellectual disability and their parents. Our hypotheses were that the intervention would improve: a) the adolescents’ and parents’ understanding of the diagnosis; b) their coping with everyday challenges; and c) their social network.

## Methods

### Design

This intervention study has a pre-post design, combining the results from three groups receiving the same intervention, but at different time points. The study also has a mixed-method design combining quantitative information from questionnaires and interviews with qualitative information from evaluation forms.

### Setting

In Norway, there are universal pediatric habilitation centers within the specialized health care system that offer multidisciplinary assessment and follow-up to children with different types of neurodevelopmental disabilities, including intellectual disability. These centers offer free of charge follow-up to all eligible children and adolescents in a geographical area. The Department of Clinical Neurosciences for Children at Oslo University Hospital provides services to children and adolescents with intellectual disability living in Oslo.

### Participants

A systematic search of the hospital records for patients born across a three-year period that had undergone neuropsychological and diagnostic evaluations, was performed. Inclusion criteria were a) having previously been given a diagnosis of mild intellectual disability, and b) being in the age range of 15–17 years at time of inclusion. Exclusion criteria were being concurrently diagnosed with a) a severe psychiatric disorder, b) autism spectrum disorder and/or c) severe behavior problems. In addition, families where parents did not have sufficient knowledge of Norwegian to follow an everyday conversation without the aid of an interpreter were not invited, as having interpreters present would alter the group dynamic. A group size of minimum six and maximum eight participants was considered ideal for this purpose (DeLucia-Waack, 2006).

Twenty-four adolescents and their parents were enrolled in three consecutive groups. The first group consisted of three boys/five girls, the second group of eight girls, the third group of five boys/three girls. Except for one participant in the second group, everyone completed the intervention (n = 23, 8 boys/15 girls). One adolescent received special needs education in regular class, the rest of the participants attended segregated special classes or special schools. All lived with their parents, and typically in two-parent families (21/23). Fifteen families were ethnic Norwegians, eight families had non-European background.

### Procedures

The intervention consisted of six weekly group sessions. The sessions were scheduled outside of work/school hours. Group cohesion was emphasized, each session starting with all participants having dinner together, and social interaction among group members was encouraged. After the meal, the adolescents participated in a group led by two clinical psychologists, and the parents in a separate group led by a clinical psychologist and a clinical social worker. Each session lasted for 2 hours, including a break with a small snack.

#### Adolescent group

This part of the intervention was inspired by the Swedish learning materials on intellectual disability called *Ninjakoll* (Olsson et al., 2007). Learning materials were adapted at a level corresponding to the participants’ cognitive capacities. Each session’s beginning and end followed the same format, and each activity had a limited duration in time. The order of activities for each session was carefully planned, with each session’s topic addressed through a variety of approaches. Overall strategies were repetition and concretization of topics and concepts, with extensive use of visual support designed to promote consolidation and increase understanding. During the sessions, topics related to the adolescents’ life situations, diagnoses, autonomy, coping and well-being were addressed, with a mix of mini-lectures, videos, group discussions, practical assignments, quiz, role play and evaluation tasks. Participants were encouraged to share their experiences related to the topic in question. While difficult topics could be discussed (e.g. bullying, social isolation), the group leaders emphasized a solution-oriented approach. Topics for the six sessions are listed in Table 1, followed by a description of the different approaches and methods applied in the adolescent group.

**Table 1.** Main topics for the six weekly sessions in the adolescent and parent groups of the psychoeducational intervention the Super Control Project.

Session	Adolescent group	Parent group
1	Get to know each other and learn about the diagnosis intellectual disability	Get to know each other and learn about the diagnosis intellectual disability
2	Learn more about intellectual disability	Network, social inclusion, recreational activities and netiquette
3	Friends and social problems, recreational activities	Upper secondary education and vocational training. Economic and practical support for adults with intellectual disability
4	Tips and tricks for everyday challenges	Family life and parenting
5	Who am I and what do I want to become?	Social welfare. Sexuality
6	Social media, netiquette, plus some time for participants wishes, e.g. about driver’s license, about having children	Transition to adulthood, including issues related to mental health and the possibility of obtaining a driver’s license

##### Mini-lectures

Brief presentations with engaging illustrations, of relevant topics (e.g., What is intellectual disability? or About driver’s license) were employed. The participants were invited to ask questions during the talks.

##### Videos

Small video clips on topics such as how the brain works, how professionals can be sure that a person has intellectual disability, how much a person with intellectual disability can learn, and how to solve everyday challenges related to issues like management of time, money and public transportation. Videos were from Ninjakoll (Olsson et al., 2007) and NRKSuper (Norwegian Broadcasting, https://nrksuper.no).

##### Practical assignments

Examples of practical assignments were Check tasks (e.g. charts with small icons where they were asked to check off tasks that they are good at and tasks which they need help with), Cartoons (e.g. where they formulated their own answers to questions related to intellectual disability) and Fill in forms for future planning related to various aspects of adult living (where they were to describe current situation, future goals and actions necessary to obtain the goals).

##### Quiz

Cards with question on topics such as relationships, social problems and social media, were used for a quiz relevant to the group members’ life situation. The adolescents took turn on picking a question card and reading the questions aloud. The alternating picking and reading of questions was planned that way to make sure everybody felt that they participated even though they didn’t know the answer to the question. Kahoot games were also applied for repetition and to keep the adolescents engaged.

##### Role play

Small role plays, performed by the group leaders wearing wigs or costumes to emphasize that it was pretend. Different scenarios and solutions to potentially difficult social situations were played out and the adolescents evaluated which alternatives they liked/disliked. The adolescents were afterwards given the opportunity to take on one of the roles to act out the alternative they liked the most, or to come up with their own way of managing the imagined situation. Role plays included social situations like: “How to explain intellectual disability to a class mate?,” “How to explain why I switch to a special ed class?,” “In which situation is it important/necessary to tell about the diagnosis and in which situation is it not?,” and “In which situation is it important/necessary to seek help and in which situation can I manage on my own?”

##### Evaluation tasks

Various types of evaluation tasks were applied throughout the intervention, to help maintain active participation and let the adolescents feel engaged without excessive exposure. Green/red cards for *yes/no* or *like/dislike* was one type of response mode used by the adolescents, e.g. in repetition tasks like true/false claims on the diagnosis (e.g. Intellectual disability can go away. Intellectual disability involves difficulty in learning. Intellectual disability is contagious). Another evaluation activity was “The hot chair”; the adolescents were seated in a circle with one free chair, and every time they agreed upon given statements, they had to change seats (for instance: “In ten years from now…(1) I have a job that I like (2) I’m the world champion in chess (3) I live in my own apartment”).

#### Parent group

The topics addressed in the parent group (see Table 1) were based on the intervention program developed by Picard et al. (2014), but were condensed into six sessions and tailored to the group. Parents were continuously informed about the topics addressed in the adolescent group, preparing them for follow-up questions from their children in between the sessions.

In the parent group, a mix of lectures from professionals working in the specialist health care services as well as invited speakers from other agencies (welfare services and educational sector) were given. The complex theme of family life and parenting was introduced by a user representative; a mother of two young adults with intellectual disability. Parents were continuously invited to ask questions, give feedback, and share their own experiences. Links to relevant websites and lists of suggested readings were provided so that parents could continue the education at their own pace and according to their personal needs. With mutual consent, the participants were provided with contact information to each other, so that they could keep in touch after the intervention had ended.

At the end of the last session, the parent and adolescent groups had a joined closing ceremony. The adolescents were given a diploma and a folder containing all the practical assignments they had been working on, as well as key points from discussions and lectures that had taken place in the adolescent group. To enhance further conversation in the family, the parents were encouraged to review the folder of practical assignments together with their child.

#### Data collection

Data were collected pre- and post-intervention without a control group, from the adolescents, the parents, and the adolescents’ primary teachers. Reports from the teachers were collected to gain information about changes in everyday functioning. Of the 23 families who completed the intervention, 20 parents filled out both questionnaires. Three of the 20 families had different responders before and after the intervention and were therefore excluded from the analyses, so that the parent questionnaire total was n = 17. Approximately half of the teachers responded to both questionnaires (n = 11). For the first group, we administered interviews with the adolescents (n = 8) pre- and post-intervention. These were conducted by a master student in special needs education under the supervision of a clinical psychologist and took place at the hospital or at the participants’ schools or homes during the weeks before and after the intervention. In all three groups, the parents and adolescents also answered evaluation forms at the end of the last group session. Twenty-one of the 23 parents, and 19 of the 23 adolescents, completed the evaluation form.

### Measures

We were unable to find instruments that fitted the target population covering the themes relevant for the intervention, and therefore we employed instruments specifically developed for this study: a questionnaire for the parents and teachers, a short modified interview for the adolescents, and semi-structured evaluation forms for the parents and the adolescents.

#### Parent and teacher questionnaire

Parents and teachers completed a 31-item questionnaire addressing adolescents’ well-being, practical, social and emotional functioning, rated on a scale from 1 (disagree) to 5 (agree). Items with a negative framing were reversed, so that higher scores meant positive outcome. We created five subscales based on thematic grouping of relevant items. Items that did not fit appropriately into subscales were discarded. The subscale *Practical skills* included the items: “Keeps track of personal belongings (clothes, stuff in his/her room, school material),” “Keeps track of appointments,” “Must be repeatedly reminded to get things done,” “Finds solutions to practical problems,” “Is helpful,” and “Takes initiative to do chores/activities.” Cronbach´s alpha for the scale, pre and post, was .74 and .71, and .60 and .62, for parents and teachers respectively. The subscale *Friends* included the items: “Has friends at school,” “Hangs out with friends after school,” “Has the belief that he/she can make new friends,” and “Is well liked.” Alpha for the scale, pre and post, was .77 and .82, and .73 and .81, for parents and teachers respectively. The subscale *Social adjustment/inclusion* included the items: “Is bullied/harassed,” “Often gets into quarrel,” and “Gets rejected by peers.” Alpha for the scale, pre and post, was .79 and .62, and .73 and .81, for parents and teachers respectively. *Insight* included the items: “Asks for help when he/she needs it,” “Has insight into own strengths,” and “Has insight into own limitations.” Alpha for the scale, pre and post, was .62 and .56, and .46 and .78, for parents and teachers respectively. The subscale *Mental health* included the items: “Seems happy and content,” “Seems to be stressed out,” “Is often sad,” “Worries a lot,” and “Gets easily annoyed/angry.” Alpha for the scale, pre and post, was .74 and .76, and .79 and .77, for parents and teachers respectively.

#### Adolescent interview

The adolescents participated in a short interview where they responded to 25 statements covering the following topics; practical everyday skills, friendships and social difficulties, self-efficacy and social support, as well as emotional and general well-being. For each statement, the adolescents decided whether they disagreed (1), sometimes agreed (2), or always agreed (3) with the statement. As a visual aid during the interview, we used an approach inspired by the *Talking Mats methodology*. Talking Mats is a low-tech communication resource that uses graphic symbols to help people with a communication difficulty understand and respond more effectively (Midtlin et al., 2015; Murphey and Cameron, 2008). Items with a negative framing were reversed, so that higher scores meant positive outcome. We created three subscales with thematic grouping of relevant items. Items that did not fit appropriately into subscales were discarded. The subscale *Practical skills* included the items: “I keep my room tidy,” “I keep my school materials in order,” “I can go shopping on my own,” “I help with household chores,” “I travel by public transportation on my own,” and “I arrive on time.” Alpha pre- and post-intervention was .58 and .59 respectively. The subscale *Social functioning* included the items: “I have good friends at school,” “I hang out with friends after school,” “I can get new friends,” “I often get into quarrel,” and “Other teenagers don’t want to be with me,” with alphas of .65 and .69 pre and post. The subscale *Mental health* included the items: “I´m happy,” “I feel stressed out,” “I feel sad,” “I often worry,” “I feel lonely,” “I often get angry,” “It feels like I never get things right,” “My life is good” and “I believe in myself.” Alpha pre- and post-intervention for this scale was .86 and .79. respectively.

#### Evaluation forms

At the end of the intervention, the parents and adolescents completed a semi-structured evaluation form. For the parents, it comprised both practical arrangements, relevance of addressed topics, and perceived usefulness of participation. For the adolescents, the evaluation form was adopted with check off alternatives (thumbs up/down/in between) for questions like “How was it to participate in the group?” and “Have you learned anything new?”, in addition to some open-ended questions on what topics they liked/disliked/missed.

### Analysis

We report differences in group mean scores pre- and post-intervention as Cohen’s *d* (the difference in mean scores divided by the average between-person standard deviation), and a hypothesis test of the change in mean score. For the hypotheses test, we use a paired sample t-test for the ease of interpretation. Due to the small sample size and violations of normality, we replicated all analyses with the non-parametric Wilcoxon signed-rank test (with substantively identical findings). Given the small sample sizes in our analyses, all hypotheses tests must be interpreted with caution.

### Ethical considerations

The project was approved by the Institutional Review Board at the hospital. Participation in the project was voluntary and the participants were advised that they could withdraw from the project at any time without explanation, as well as assurance that withdrawal would not interfere with future treatment at the hospital. Parents and adolescents signed informed written consent. The majority of adolescents enrolled in the groups did not know about the fact that they had the diagnosis of mild intellectual disability beforehand, which made it necessary to provide them with adapted, diagnosis-related information prior to the intervention. This was conducted as a one-on-one consultation with a clinical psychologist, and with one parent present.

## Results

### Questionnaire/interview

Descriptive statistics pre- and post-intervention for all scales are in Table 2. There were improvements on all subscales from pre- to post-intervention, on parent, teacher, and adolescent reports. For parent reports, the effect-sizes (Cohen’s *d*) varied from .11 to .36, with statistically significant changes for *Practical skills* (*p* < .05) and *Mental health* (*p* < .10). The effect sizes are in nominal terms slightly larger for the teachers, but show a greater variability ranging from .04 to .43, with statistically significant changes in *Social adjustment/inclusion* (*p* < .01). The adolescents themselves reported on average considerable changes, with effect sizes ranging from .63–.93, all of them significant at a *p* < .10-level.

**Table 2.** Descriptive statistics and test of change in pre- to post-scores on questionnaire answered by parents and teachers and adolescent interview.

	Pre M(SD)	Post M(SD)	Cohen’s *d*	df	t-value
Parents (n = 17)					
Practical skills	3.07(0.73)	3.28(0.53)	0.34	16	2.60*
Friends	3.34(0.22)	3.50(0.21)	0.18	16	1.26
Social adjustment/inclusion	3.80(0.96)	3.90(0.78)	0.11	16	0.46
Insight	3.37(0.68)	3.53(0.57)	0.25	16	1.29
Mental health	3.56(0.58)	3.79(0.70)	0.36	16	1.77^+^
Teachers (n = 11)					
Practical skills	3.56(0.48)	3.73(0.26)	0.43	10	1.23
Friends	3.77(0.84)	3.90(0.76)	0.16	10	0.72
Social adjustment/inclusion	3.91(0.87)	4.21(0.70)	0.38	10	3.19**
Insight	3.18(0.58)	3.39(0.65)	0.34	10	1.55
Mental health	3.47(0.55)	3.50(0.51)	0.04	10	0.17
Adolescents (n = 8)					
Practical skills	2.55(0.25)	2.76(0.20)	0.93	7	3.31*
Social functioning	2.38(0.29)	2.65(0.37)	0.83	7	3.37*
Mental health	2.38(0.39)	2.58(0.20)	0.62	7	2.21^+^

*Note:* Parents and teachers reported on a Likert scale ranging from 1–5. Adolescents responded to interview probes with three response options. **p < .01, *p < .05, ^+^p < .10

### Evaluation forms

#### Intervention contents and implementation

All parents found information provided prior to enrollment to be comprehensive and sufficient. The dinner arrangement was perceived both as welcomed in an otherwise tight schedule, and as an icebreaker for social interaction. The quality of the invited speakers, as well as the format of the sessions, were positively rated. The topics covered were in general rated by the parents as highly relevant and useful (see Table 3). The least appreciated session was on the topic of driver´s license, possibly because the majority of the parents did not envisage that their adolescent should become a driver. Twelve parents commented that they appreciated the generous climate that was created in the group; that there was room to share joy and sorrow, and that it gave them opportunity to learn from other parents’ experiences and ways of coping. Social support and time to discuss relevant topics with both professionals and other parents in the same situation were described as reassuring and validating. Moreover, in both evaluation forms and through direct communication, parents spontaneously stated that an intervention like this was something that they had missed, and that they would recommend to other parents.

**Table 3.** Parents’ evaluation of addressed topics in the Super Control Project (n = 21).

	Obs	M(SD)
Learn about Intellectual Disability	19	4.32(0.82)
Network, social inclusion, recreational activities and netiquette	21	4.43(0.60)
Upper secondary education, vocational training/Economic and practical support	20	4.65(0.59)
Family life and parenting	21	4.43(0.68)
Social welfare	19	4.74(0.56)
Sexuality	18	4.06(1.06)
Mental health	20	4.75(0.44)
Driver’s license	19	3.55(1.23)

*Note:* Perceived level of relevance on a Likert scale ranging from “less relevant” (1) to “very relevant” (5)

#### Perceived usefulness

The parents’ and adolescents’ evaluations of perceived usefulness suggest that the intervention had a positive impact with regard to both understanding of the diagnosis, managing of everyday challenges and social networking.

##### Understanding of diagnosis

Nineteen of the 21 parents reported that the intervention had given them increased insight into the diagnosis, and better understanding of its implications. Eighteen parents perceived the intervention to have been useful for their adolescents’ understanding of intellectual disability. Specifically, there were responses such as: “He/she has become more aware of his/her diagnosis”, and “It has raised a lot of thoughts about who he/she is.” One parent noted the possibility that participation in the intervention might had led the adolescent to a more pessimistic view on life. Four parents commented that the adolescents gained self-esteem from participation. Six parents stated that participation in the intervention yielded new topics of conversation in the family, and nice talks with their adolescents.

In interviews, the adolescents reported improved knowledge on intellectual disability and related topics. Specifically, some statements signaled acceptance and empowerment; “I’ve been thinking that now, I dare to tell people that I have it [intellectual disability], more than just to hide it from friends and family, because if I just tell them right out, they will respect me” and “It [intellectual disability] doesn’t show on the outside. We’re like, not that super special because of it, we’re like normal people. Others should accept that.” One of the adolescents expressed anger with her parents for knowing about the diagnosis for several years without telling her and imagined that if she *had* known earlier it would have made it easier to reconcile with previous life events such as having to switch from a regular to a special educational class.

##### Managing of everyday challenges

Parents reported that the intervention provided them with useful information on how and where to seek help, and that it was reassuring to get an overview of the available support services. Seven parents remarked that participating in the intervention had increased awareness of planning for the future and made them more proactive in this regard. Specifically, there were responses like: “I feel much better prepared for the adult life of my child.” Three parents stated that the intervention had made their child more dedicated to master everyday skills. The adolescents also reported benefits in terms of managing everyday challenges, for instance there were responses such as: “Things will work out better for me now that I’ve heard about the others’ experiences” and “It’s nice to know that I don’t have to worry about management of money, that there’s someone who can help me out.” Some adolescents also appreciated the focus on adult life and autonomy.

##### Social network

It was evident that the participants established new friendships through the intervention. Of the 19 adolescents whom completed the evaluation form, 17 replied “thumbs up” to the question “How was it to spend time with the other group members?.” There were social interactions among several of the adolescents in between sessions, as well as after the intervention had ended. The parents also expressed an intention to keep in touch.

## Discussion

The present study describes a structured psychoeducational group intervention for adolescents with mild intellectual disability and their parents and evaluates it’s usefulness. The results were encouraging, suggesting positive impacts with regard to understanding of the diagnosis and managing of everyday challenges and social networking during this crucial time period of transitioning to adulthood.

All the participating families, but one, completed the intervention, thus group attendance and dropout as indicators gave preliminary evidence of the acceptability of the program. The evaluation indicated satisfaction with the practical implementation of the intervention, that the topics were relevant to their personal situation, and that the group setting offered an opportunity for sharing personal experiences and for social network and support. Parent/teacher questionnaire and adolescent interview showed improvements on all subscales from pre to post intervention, some of which were statistically significant.

One of the most commonly cited needs for parents of children with developmental disabilities is information about diagnosis, support and services (Ellis et al., 2002). Consistent with results of multi-family psychoeducational group interventions for parents of individuals with intellectual disability (Picard et al., 2014), our findings suggest benefits in terms of parents becoming more knowledgeable about the diagnosis, and thus increasing their awareness of planning the future and making them more proactive in seeking support. Our intervention differs from others in that it simultaneously aims directly at educating the adolescents themselves along with their parents. All patient groups, including people with intellectual disability, are entitled to information about their diagnosis. Preliminary findings from our study yielded promising results, suggesting that adolescents with limited cognitive resources indeed can benefit from an adapted psychoeducational approach, both with regard to learning about the diagnosis and related issues, and with regard to the social benefits from receiving such type of education in a group setting.

Executing a psychoeducational group intervention for adolescents with mild intellectual disability poses some challenges, both in terms of the need for thoroughly planning the structure and content of each session, as well as providing thoughtful leadership for enhancement of group dynamics and social exchange between participants. In addition, the stigma associated with the diagnosis represents a challenge in terms of conveying an empowering approach to the subject matter. Feedback from the adolescents in our groups nevertheless indicated emerging acceptance and empowerment, opportunity to learn from each other, and appreciation of the positive and generous tone that was created in the group. Jones et al. (2014) highlighted the importance of promoting proactive conversations about disability between parents and adolescents with intellectual disability. We believe that the simultaneousness of our intervention design might represent a key-factor in this regard. Educating parents and adolescents at the same time enhance further exchange in the family and can thus serve as the beginning of a recognition and consolidation process of the diagnosis and its implications. Contrary to other studies (e.g. González-Fraile et al., 2019), we had no problem with recruitment and the drop-out rate was low. There is reason to believe that the practical arrangements we offered, with simultaneous sessions that were scheduled after school/work hours and that included dinner serving, met our participants’ needs, and thus contributed to group attendance.

### Limitations

Several factors placed limitations on our study. Most notably, it was limited by a small number of participants, and lack of a control group. The lack of a control group implies that it is not possible to attribute observed improvements to the intervention. A randomized, case-controlled study design would be the preferred method to assess the impact of our intervention. Further, the present study had only one time of data-collection following the intervention, and the intervention, consisting of six weekly sessions, was relatively brief in nature. We need to consider long term follow up to monitor potential positive changes after a period of consolidation in the family, but also to monitor potential negative changes related to the adolescents’ increased insight. A six-week intervention period was what was practically feasible in our clinical setting, but might be too short to achieve significant and persistent changes. A viable intermediate solution would be to further facilitate the participants to continue to meet as a social support group, by providing them with meeting facilities and a pre-planned schedule.

This study focuses on a critical gap in research and practice for adolescents with intellectual disability. The scarcity in the literature of psychoeducational interventions for the group, highlights the undeniable need for additional psychoeducational studies. The use of group-based psychoeducational interventions has demonstrated positive changes for people with other types of chronic health conditions and varied life challenges (Lukens and McFarlane, 2004). However, many issues remain unanswered regarding the usefulness of such interventional approaches aimed directly at adolescents with intellectual disability. More work is needed to examine which psychoeducational content components would most likely have a meaningful impact on various aspects of their challenging transition to adulthood.

## Conclusion

Adding to the emerging literature on ways to support adolescents with developmental disabilities and their parents, our group-based psychoeducational intervention yielded promising results. The intervention seemed to fit the participants’ needs and abilities. It does not require extensive training by the group leaders and can easily be implemented in other habilitation centers. Group-delivered interventions are more cost-effective than individually delivered support, and the group format enhances social network and support. This study encourages further implementation and rigorous evaluation.
